# Giant ovarian serous cystadenoma in a postmenopausal woman: a case report

**DOI:** 10.4076/1757-1626-2-7875

**Published:** 2009-07-23

**Authors:** Vellanki Venkata Sujatha, Sunkavalli Chinna Babu

**Affiliations:** Department of Obstetrics and Gynaecology, Kamineni Institute of Medical Sciences SreepuramNarketpally, Nalgonda - 508254, Andhra PradeshIndia

## Abstract

A case of 66-year-old South Indian post menopausal woman presenting a giant ovarian serous cyst adenoma weighing 23 kg is reported here. A 66-year-old woman was referred to our clinic from a local medical center. When she was seen first at our outpatient clinic, she had gross abdominal distension since 2 years and she was unable to walk. On abdominal ultrasound, a giant cyst was found which encompassed the whole abdomen. At laparotomy, a giant, totally cystic, vascularized and smooth mass attached to the right ovary was encountered. Staging laparotomy was performed. On the postoperative tenth day, she was discharged without any problem. Her pathology report disclosed a 60×47×30 cm serous cyst adenoma weighing 23 kg. This is the largest ovarian cyst that ever reported from our hospital and one of the largest among the reported cases in the literature.

## Introduction

Ovarian neoplasms may be divided by origin cell type into three main groups: epithelial, stromal and germ cell. Taken as a group, the epithelial tumors are by far the most common type. The single most common benign ovarian neoplasm is the benign cystic teratoma; however, according to some studies it is serous cyst adenoma [1].

## Case presentation

A 66 year old South Indian post menopausal woman presented at the outpatient department with gradually increasing abdominal swelling first noticed 2 years back. Swelling was accompanied by vague pain all over the abdomen for 6 months and increasing in size from four months. Due to the huge mass she was unable to walk and had anorexia. There was no history of colicky pain, fainting attacks, vomiting or other gastrointestinal disturbances. Her bowel and bladder habits were normal. There was history of generalised weakness. She had no serious illness or operation before. She is P6L5D1, married since 47 years and attained menopause 20 years back. There was no family history of malignancies.

On general examination, she was thin built and undernourished. She weighed 55 kilograms. Pallor was present; she was normotensive and had bilateral pitting pedal odema. On abdominal examination, abdomen was grossly distended, engorged veins present, Fluid thrill was present. Abdominal girth at the level of the umbilicus was 42 inches. Bowel sounds were heard in the flanks. Vaginal examination showed cystocoele, cervix was deviated to right flushed with vault and both the fornices were full. Pre operative investigations including liver and renal functions were normal. Ca125 was 46.61 ng/ml. Chest X-Ray showed cardiomegaly and elevation of both domes of diaphragm.

**Figure 1.**
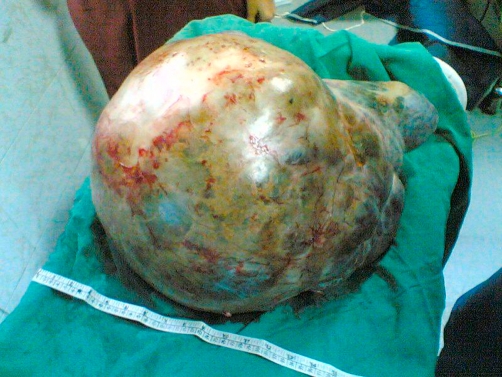
Giant right ovarian cyst measuring 60 × 47 × 30 cm has been removed intact.

On ultrasound examination, uterus was atophic and seen separate from the mass. Large cystic mass seen occupying the whole abdomen and with multiple septations. Abdominal organs were compressed by the mass. Right side pelvicalceal system dilatation was seen. A differential diagnosis of gross ascites and large ovarian cyst (malignant) were made. Magnetic resonance imaging confirmed the diagnosis of right sided ovarian cyst with multiple thin septations. Staging laparotomy was done. Abdomen was opened by a vertical midline incision extending above the level of the umbilicus. A tense smooth surfaced cystic mass measuring 60 × 47 × 30 cm extending up to then undersurface of the diaphragm was delivered out (Figure 1). The mass originated from the right ovarian region. The right ovary was included in the mass. The right fallopian tube was thinned out, adherent, and stretched over the surface of the cyst. There was no free fluid in the abdomen. The cyst was excised intact. Total abdominal hysterectomy with salpingoopherectomy was done. Both tubes and ovaries, and the uterus were healthy. On histopathology, the section of the cyst was thin walled and lined by columnar epithelium and stroma containing spindly fibroblasts. Nuclear atypia and increased mitotic index were not observed suggestive of benign serous cystadenoma. The sections from the uterus, tubes and the ovaries were normal. Postoperative period was uneventful and patient was discharged on tenth day. Patient did not have any urinary problem and the hydronephrotic changes in the kidneys gradually resolved after 3 months.

## Discussion

Giant ovarian serous cyst adenoma of such a huge size is a rare finding. The most remarkable descriptions of large ovarian cysts are those of Spohn, who in 1922 reported one that weighed 148.6 kg (328 lb), and of Symmonds, who in 1963 reported encountering one that weighed 79.4 kg (175 lb) Such descriptions were among the curiosities reported in the 19th and early 20th centuries. They have become rarer as imaging modalities improve and diagnoses are made earlier [2-4].

Our patient had delayed reaching the hospital due to financial constraints and did not want to take the risk of surgery and approached us only after it was totally debilitating and she was totally bed ridden. Ovarian epithelial tumors comprise about half of all ovarian tumors, accounting for about 40% of benign tumors and 86% of malignant tumors. Benign serous tumors include cyst adenomas, adenofibromas, cyst adenofibromas and surface papillomas. These tumors are common, accounting for about 25% of all benign ovarian neoplasms and 58% of all ovarian serous tumors. The serous tumors are bilateral in about 10% of cases of all serous tumors, about 70% are benign, 5-10% have borderline malignant potential and 20-25% are malignant, depending largely on the patient’s age. They tend to be multilocular but unilocular serous cyst adenomas are not uncommon. They present grossly as large as 35 cm in diameter, spherical or ovoid masses, like the one reported here [1].

## Conclusions

This is the largest ovarian tumour ever reported in our hospital and is one of the largest tumours reported in literature.

## Abbreviation

P6L5D1: para 6 living 5 death 1

## References

[bib-001] Mülayim B, Gürakan H, Dagli V, Mülayim S, Aydin O, Akkaya H (2006). Unaware of a giant serous cyst adenoma: a case report. Arch Gynaecol Obstet.

[bib-002] S, Shvartzman P (1994). Giant ovarian cyst mimicking ascites. J Fam Pract.

[bib-003] Mukhopadhyay Sima (2006). Giant paraovarian cyst. J Obstet Gynecol India.

[bib-004] Young TH, Lee HS (2008). Images in clinical medicine. Giant Ovarian Cyst N Engl J Med.

